# FungalRV: adhesin prediction and immunoinformatics portal for human fungal pathogens

**DOI:** 10.1186/1471-2164-12-192

**Published:** 2011-04-15

**Authors:** Rupanjali Chaudhuri, Faraz Alam Ansari, Muthukurussi Varieth Raghunandanan, Srinivasan Ramachandran

**Affiliations:** 1 G.N Ramachandran Knowledge Centre for Genome Informatics, Institute of Genomics and Integrative Biology, Mall Road, Delhi 110007, India

## Abstract

**Background:**

The availability of sequence data of human pathogenic fungi generates opportunities to develop Bioinformatics tools and resources for vaccine development towards benefitting at-risk patients.

**Description:**

We have developed a fungal adhesin predictor and an immunoinformatics database with predicted adhesins. Based on literature search and domain analysis, we prepared a positive dataset comprising adhesin protein sequences from human fungal pathogens *Candida albicans, Candida glabrata, Aspergillus fumigatus, Coccidioides immitis, Coccidioides posadasii, Histoplasma capsulatum, Blastomyces dermatitidis, Pneumocystis carinii, Pneumocystis jirovecii and Paracoccidioides brasiliensis*. The negative dataset consisted of proteins with high probability to function intracellularly. We have used 3945 compositional properties including frequencies of mono, doublet, triplet, and multiplets of amino acids and hydrophobic properties as input features of protein sequences to Support Vector Machine. Best classifiers were identified through an exhaustive search of 588 parameters and meeting the criteria of best Mathews Correlation Coefficient and lowest coefficient of variation among the 3 fold cross validation datasets. The "FungalRV adhesin predictor" was built on three models whose average Mathews Correlation Coefficient was in the range 0.89-0.90 and its coefficient of variation across three fold cross validation datasets in the range 1.2% - 2.74% at threshold score of 0. We obtained an overall MCC value of 0.8702 considering all 8 pathogens, namely, *C. albicans, C. glabrata, A. fumigatus, B. dermatitidis, C. immitis, C. posadasii, H. capsulatum *and *P. brasiliensis *thus showing high sensitivity and specificity at a threshold of 0.511. In case of *P. brasiliensis *the algorithm achieved a sensitivity of 66.67%. A total of 307 fungal adhesins and adhesin like proteins were predicted from the entire proteomes of eight human pathogenic fungal species. The immunoinformatics analysis data on these proteins were organized for easy user interface analysis. A Web interface was developed for analysis by users. The predicted adhesin sequences were processed through 18 immunoinformatics algorithms and these data have been organized into MySQL backend. A user friendly interface has been developed for experimental researchers for retrieving information from the database.

**Conclusion:**

FungalRV webserver facilitating the discovery process for novel human pathogenic fungal adhesin vaccine has been developed.

## Background

As cases of immunosuppression rise, the spectrum of fungal pathogens is increasing thus posing a serious threat to human health. In the USA and in most European countries infection due to Candida species have become very common [1]. Amongst the *Candida spp*, *C. albicans *and *C. glabrata *account for approximately 70-80% of Candida species recovered from patients with candidemia or invasive candidiasis [2,3]. Another pathogenic fungi, *A. fumigatus *is the most common life-threatening aerial fungal pathogen which primarily affects the lungs. In severe invasive aspergillosis caused mainly in immunocompromised individuals, the fungus can transfer from lungs through blood stream to brain and other organs. This condition of invasive aspergillosis is often associated with significant mortality and morbidity [4,5]. In addition, certain non-life-threatening superficial and respiratory infections caused by dimorphic pathogenic fungi like *C. immitis, H. capsulatum, P. brasiliensis *and *B. dermatitidis *impose significant restrictions on patients, resulting in a reduced quality of life. In some cases these infections may turn to life threatening specially in immunocompromised patients, where the infection spreads beyond the respiratory system to other parts of the body [6-10]. Another fungal infection *Pneumocystis *pneumonia (PCP) or pneumocystosis caused by unusual unicellular fungi *Pneumocystis jirovecii *(formerly called *Pneumocystis carinii*) is the most common opportunistic infection in persons with HIV infection [11].

It is challenging to identify candidates for vaccines in case of fungal infections because of their occurrence in immunocompromised or otherwise debilitated host. Yet it is being realized that either a preventive or therapeutic vaccine could be useful for at-risk patients [12,13].

Adhesins are important virulence factors used by pathogens during establishment of infection. Therefore, targeting the adhesins in vaccine development can help efficiently combat fungal infections by blocking their function and preventing adherence to host cell [14]. A few vaccine formulations using adhesins as immunizing agents and are under evaluation include agglutinin-like sequence proteins in *Candida albicans*[15,16], BAD-1(WI adhesin) protein in *Blastomyces dermatitidis*[17,18], 43 kDa glycoprotein in *Paracoccidioides brasiliensis*[19,20] and spherule outer wall glycoprotein in *Coccidioides immitis*[21,22]. Among these, the spherule outer wall glycoprotein in *Coccidioides immitis *has undergone trial in humans, while others have proved their efficacy in mouse experimental models.

Most fungal adhesins have a general structure consisting of an N-terminal carbohydrate or peptide-binding domain, central Ser-Thr rich glycosylated domains and C-terminal region mediating covalent cross-linking to the wall through modified glycosylphosphatidylinositol (GPI) anchors [23,24]. Others such as WI-1/Bad1 adhesin (from *B. dermatiditis*), Int1p adhesin (from *C. albicans*) do not conform to this general structure thereby causing difficulty in their identification. Using similarity search approach, Weig et al. (2004) and Butler et al. (2009) identified adhesins and GPI-anchored proteins in certain fungal pathogens [25,26]. These efforts can be complemented using machine learning techniques trained on compositional properties in the identification of novel adhesins because in principle, this approach allows development of a non-homology composition based method. The similarity based approach in principle enable identifying members of related family whereas the non-homology composition based method has potential to identify other novel members. Algorithms based on compositional properties for adhesin identification in different pathogenic species such as Plasmodium and bacteria have been useful [27,28], encouraging us to attempt to develop a similar method for fungal species. Here, we present an algorithm developed by using Support Vector Machine trained through a combination of 3945 compositional properties for classifying human pathogenic fungal adhesins and adhesin like proteins. The predictions from these algorithms can be integrated with the immunoinformatics algorithms to facilitate rational vaccine development using reverse vaccinology [29,30]. The immunoinformatics data on the predicted fungal adhesins and adhesin like proteins are also organized for easy analysis and retrieval. These resources are made available through a user friendly interface FungalRV.

## Construction and Content

### Dataset Preparation

#### Positive Dataset

Through literature survey we collected known human pathogenic fungal adhesin protein sequences from *C. albicans, C. glabrata, A. fumigatus, B. dermatitidis, C. immitis, C. posadasii, H. capsulatum, P. brasiliensis, P. jirovecii *and *P. carinii*. In *C. glabrata *proteins having PA14 and GLEYA adhesin domain were also included [31,32]. Sequences were collected from the National Center for Biotechnology Information (NCBI) [33], Candida Genome Database (CGD) [34] and Swiss-Prot Databases [35].

#### Negative Dataset

Protein sequences which are not likely to be on the surface, or associated with adhesion were collected from NCBI, CGD and Swiss-Prot using keywords 'dehydratase', 'ribosomal protein', 'kinase', 'polymerase', 'acyl-CoA synthase', 'decarboxylase', and 'hydrolase'. Poorly annotated sequences were not considered. Pfam domain search was performed on negative dataset sequences. The results were analyzed exhaustively and any extracellular location associated domain containing protein sequence in the negative dataset was excluded. 'See additional file 1: Pfam domain search result of negative dataset'.

#### Proteomes

Proteomes of freely available fungal pathogens were sourced from various databases listed in Table 1. [36-38]

**Table 1:** List of databases from which the human pathogenic fungal proteomes were sourced.

Species	Source	Reference
*Candida albicans *(21^st ^assembly)	Candida Genome Database	[34,26]

*Candida glabrata*	Genolevures	[36,26]

*Aspergillus fumigatus*	J. Craig Venter Institute	[37]

*Coccidioides immitis *RMSCC 2394	Broad Institute	[38,26]

*Coccidioides posadasii *Silveira	Broad Institute	[38,26]

*Histoplasma capsulatum *Nam1	Broad Institute	[38,26]

*Paracoccidioides brasiliensis *Pb01	Broad Institute	[38,26]

*Blastomyces dermatitidis *SLH14081	Broad Institute	[38,26]

*Candida dubliniensis*	Sanger Institute	[26]

*Candida tropicalis*	Broad Institute	[38,26]

*Candida parapsilosis*	Broad Institute	[38,26]

*Candida lusitaniae*	Broad Institute	[38,26]

*Candida guilliermondii*	Broad Institute	[38,26]

#### Rendering datasets nonredundant

The stringent criterion (S = 100, L = 1, b = T) specified in the BLASTCLUST computer program was used to identify redundancy. Redundant entries were removed using Shell scripts. The final positive dataset had 101 non redundant adhesin protein sequences and the negative dataset had 2644 non redundant protein sequences.

### Compositional Attributes Used

After several attempts using different combinations of compositional properties, we finally settled on the following:

#### Amino acid frequencies

Xi is the counts of i^th ^amino acid in the sequence, i = 1, ..., 20 for each of the amino acid type and L is the length of the protein. There are 20 possible values for fi(a) for 20 amino acids.

#### Multiplet frequencies

Multiplets are defined as homopolymeric stretches (X)n where X is the amino acid and n (integer) ≥ 2 [39]. After identification of all the multiplets, the frequencies of the amino acids in the multiplets were computed as follows:

Xmi is the counts of i^th ^amino acid occurring as multiplet. There are 20 possible values for fi(m) for each of the 20 amino acids; and L is the length of the protein.

#### Dipeptide frequencies

The frequency of a dipeptide (i, j),

where i, j = 1...20 for each of the 20 amino acids and L is the length of the sequence. The best dipeptides discriminators between positive and negative sets were identified with the help of Welch's t test in R statistical software (ver 2.9.2) [40]. Top 247 dipeptides were selected at cutoff significance at *P-value *< 0.001.

#### Tripeptide frequencies

The frequency of a tripeptide (i, j, k),

where i, j, k = 1-20. The best tripeptides discriminators between positive and negative sets were identified with the help of Welch's t test in R statistical software (ver 2.9.2) [40]. Top 3653 tripeptides were selected at cutoff significance at *P-value *< 0.001.

#### Hydrophobic Composition

Each amino acid is given a hydrophobicity score between +4.5 and -4.5 according to Kyte and Doolittle hydrophobicity scale [41]. A score of +4.5 is the most hydrophobic and a score of -4.5 is the most hydrophilic. The hydrophobic amino acids with positive score A, M, C, F, L, V, I were selected. The frequency of hydrophobic amino acids (A, M, C, F, L, V, I) is given by,

where L is the length of the protein. Furthermore, information on the characteristics of the distribution of these amino acids in a given protein sequence was obtained by computing the moments of the positions of the occurrences of these amino acids. The general expression to compute moments of a given order; say 'r' is,

Mr = r-th order moment of the positions of hydrophobic amino acids

Where, 

*X*_*m *_is the mean of sequence positions of all hydrophobic amino acids, *Xi *is the sequence position of the i^th ^hydrophobic amino acid where i is any of the 7 hydrophobic amino acids A, M, C, F, L, V, I; and N is the total number of hydrophobic amino acids in the sequence and r is from 2-5. The values of the r^th ^order moments were downscaled to smaller decimal values by dividing by (1000)^r ^while preparing the feature input to SVM.

Thus, a total of 3945 compositional properties included amino acid frequencies of 20 from amino acids, 247 selected dipeptide frequencies, 3653 selected tripeptide frequencies, 20 amino acid multiplets frequencies, frequency of the hydrophobic amino acids and moments of hydrophobic amino acid distribution of order from 2-5.

Each sequence is represented by 3945 features. Programs in C language were written to calculate these compositional properties. These compositional properties will serve as an input for the machine learning algorithm SVM.

### SVM implementation

SVM is a supervised machine learning algorithm first introduced by Vapnik[42] used for problems involving classification and regression. In this study SVM was implemented using SVM^light ^package written and distributed by Thorsten Joachims[43]. This package has two modules svm_learn and svm_classify.

svm_learn: svm_learn is used prepare models(classifiers) built by learning from the training sets- positively and negatively labeled datasets labeled +1 and -1 respectively.

svm_classify: svm_classify is used by the models(classifiers) generated by svm_learn to classify the test set sequences (labeled 0).

#### Training and Testing process

The model (classifiers) are built using svm_learn module of SVM^light^. The training set was a file containing positively and negatively labeled samples labeled +1 and -1 respectively mixed in alternating order. Each positive sample corresponding to a positive protein sequence had +1 label followed by 3945 compositional properties. Similarly each negative sample has -1 label followed by 3945 compositional properties.

We have used two types of kernel functions, the polynomial function and the radial basis function (RBF). For polynomial kernel, all the SVM parameters were set to default, except d and C, the trade-off between training error and margin. The scalable memory parameter (m) was fixed to 120. The values for d and C were incremented stepwise through a combination of 1, 2, 3, 4. . .to . . . 9 for d, and 10^-5 ^to. . .10^15 ^for C. For the RBF kernel, the parameter gamma g and C were incremented stepwise through a combination of 10^-15 ^to . . .10^3 ^for g, and 10^-5 ^to. . .10^15 ^for C. Svm_light was provided with these parameters along with the input training set and by varying these parameter values total 588 models are generated.

Subsequently each model was input to svm_classify to classify the test set sequences. The test set is a file containing positively and negatively labeled samples labeled 0 mixed in alternating order. The 3945 features of these samples were classified and the result is a numerical value for every sample. This numerical value above set threshold value of 0.0 is indicative of the sequence being classified as positive label or negative. This prediction is compared to our known knowledge of test set and performance of the model is evaluated.

#### Threefold Cross Validation

**Figure 1:**
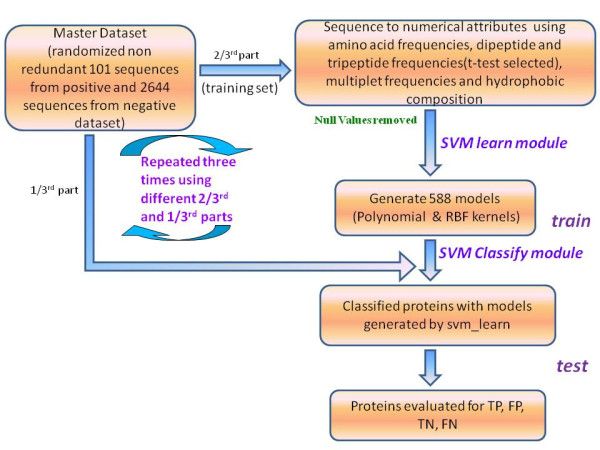
**Support Vector Machine (SVM) run flowchart**. SVM was trained and tested following this flow process, and the best classifiers were selected.

In order to obtain good performing models, threefold cross validation was done. Both positive and negative datasets were randomized 1000 times and divided into three parts, each having nearly equal number of proteins. The positive and negative subsets were merged to obtain three subsets. Then training and testing is conducted three times, each time using two subsets for training and the remaining third set for testing. Thus, each time, the testing is done on those proteins that are not a part of the training set (Figure 1). The assessment results of each test was carried out by computing the Mathews Correlation Coefficient (MCC values) [44] for each set of parameters, averaged over the three test sets and ranked in descending order of average MCC.

#### Performance evaluation

To evaluate the performance of the algorithm, specificity (SP), sensitivity (SN), accuracy (ACC) and MCC were computed as per the following formulas:

Accuracy given by

MCC given by

where TP is true positives; TN is true negatives; FP is false positives; FN is false negatives.

All evaluations were carried out at a base cutoff value of 0.0 as discriminator between positive and negative samples. This entire process was automated using perl scripts. Subsequently, coefficient of variation (CV) of MCC of each model across the three subsets was also calculated. In the next step, the models were arranged in descending order of MCC in each of the three subsets and the models with high average MCC value [0.831-0.919 (maximum)] and low CV (≤5%) were shortlisted.

### Performance Check on Human pathogenic fungal species

The performance of each of these shortlisted models was evaluated on the entire proteomes of the eight fungal pathogens by testing their ability to identify known adhesins. We finally selected the best three models for the "Fungal RV adhesin predictor". These models along with the parameters are listed in Table 2. The final score is defined as F_prediction _given by max{score(F_470a_)∪score(F_470b_)∪ score(F_449c_)} where max means maximum value in the expression. This produced minimal false positives.

**Table 2:** Parameter Sets and Performances of three Selected Models to Identify Fungal Adhesins and Adhesin-Like Proteins in human pathogenic fungal species.

Best model(classifier) selected	Kernel Type	Parameters	Performance of best model (MCC) in the selected subset	Mean MCC for parameters accoss three subsets	CV for parameters accross three subsets	Accuracy
470a	RBF	g = 0.01 C = 100	0.9189	0.8981	2.74%	99.45%

470b	RBF	g = 0.01 C = 100	0.9044	0.8981	2.74%	99.34%

449c	RBF	g = 0.001 C = 100	0.8876	0.8922	1.20%	99.23%

### Receiver operating characteristic Curve

**Figure 2:**
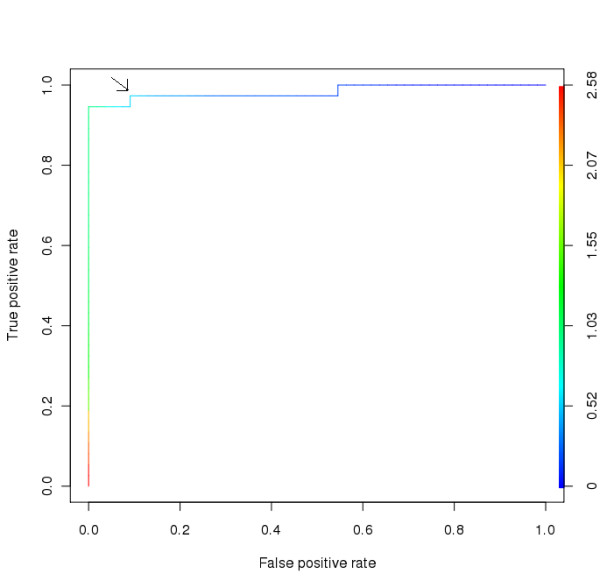
**Receiver operating characteristic curve**. The selected optimal threshold value (marked by arrow) for "FungalRV adhesin predictor" is shown.

The Receiver operating characteristic Curve (ROC curve) was made from the result of "FungalRV adhesin predictor" run on the proteomes of eight human fungal pathogens. Proteins above the default threshold score of 0.0 were examined. Known adhesins were marked as true positives while proteins with probability to function intracellularly were marked false positives. The R software package ROCR was used to make the ROC curve [45]. The best threshold inferred from the ROC curve is 0.873. However we observed that this is too stringent and may miss prediction of many adhesins. Therefore the next point in ROC curve at threshold value of 0.511 was selected. Using this threshold, the algorithm is able to achieve a sensitivity of 100% for all human pathogens except in *P. brasiliensis *wherein a sensitivity of 66.67% was achieved. The overall MCC value of 0.8702 was achieved considering all 8 pathogens (Figure 2, Table 3).

**Table 3:** Summary of predictions by FungalRV adhesin predictor using optimal threshold of 0.511.

Species	Number of Proteins above threshold	Number of Known Adhesins in proteome	Number of adhesins identified (Sensitivity)	Number of hypothetical Proteins	Number of false positives
*A. fumigatus*	38	2	2(100%)	20	0

*C. albicans*	81	14	14(100%)	0	1

*C. glabrata*	62	20	20(100%)	0	0

*B. dermatitidis*	33	1	1(100%)	10	2

*C. immitis*	23	1	1(100%)	8	0

*C. posadasii*	27	1	1(100%)	13	1

*H. capsulatum*	21	1	1(100%)	6	1

*P. brasiliensis*	27	3	2(66.67%)	11	0

### Performance Check on other fungal species

Though our server focuses on human fungal pathogens adhesin prediction, we also checked its performance on a test set of fungal species not pathogenic for human. This test set of proteins was prepared from the Swiss-Prot and the NCBI database by using search keywords "fungi" and "adhesin", ''flocculin", "agglutinin". After removing the sequences corresponding to the human fungal pathogens we obtained 74 sequences from *Pichia spp, Debaryomyces spp, Saccharomyces spp, Lachancea spp, Schizosaccharomyces spp, Kluyveromyces spp, Zygosaccharomyces spp, Neosartorya spp, Talaromyces spp, Botryotinia spp, Nectria spp, Metarhizium spp, Verticillium spp, Emericella spp, Vanderwaltozyma spp, Beauveria spp, Trichoderma spp, and Magnaporthe spp*. In this case, a different combination of models of high MCC and low coefficient of variation appear appropriate in identifying 61 of 74 adhesins and thus giving a high sensitivity of 82.43%. The F_prediction _for this case is given by max{score(F_26a_)score(F_470b_) score(F_6c_)} where max means maximum value in the expression. These models along with their parameters are listed in Table 4.

**Table 4:** Parameter Sets and Performances of three Selected Models to Identify Fungal Adhesins and Adhesin-Like Proteins in other fungi (not pathogenic to human).

Best model(classifier) selected	Kernel Type	Parameters	Performance of best model (MCC) in the selected subset	Mean MCC for parameters accoss three subsets	CV for parameters accross three subsets
26a	polynomial	d = 2 c = 0.1	0.9019	0.89	3.24%

470b	RBF	g = 0.01 c = 100	0.9044	0.8981	2.74%

6c	polynomial	d = 1 c = 1	0.9044	0.8945	0.9%

### Immunoinformatics Data

#### Database architecture

Protein sequences of known fungal vaccine candidates and of 307 predicted adhesins and adhesin like proteins were analyzed with 18 immunoinformatics algorithms displayed in Table 5. The ORF identification tags (ORF ID) assigned to proteins of fungal pathogens as given in the respected database repositories mentioned earlier were used as primary keys.

**Table 5:** Algorithms used to analyse predicted adhesins for Immunoinformatics.

*Algorithm*	*Principle*	*Reference*
1. BLASTCLUST	Clusters protein or DNA sequences based on pairwise matches found using the BLAST algorithm in case of proteins or Mega BLAST algorithm for DNA.	[60]

2. OrthoMCL	OrthoMCL software was used to cluster proteins based on sequence similarity, using an all-against-all BLAST search of each species' proteome, followed by normalization of inter-species differences, and Markov clustering.	[61]

3. BetaWrap	Predicts the right-handed parallel beta-helix supersecondary structural motif in primary amino acid sequences by using beta-strand interactions learned from non-beta-helix structures.	[62]

4. Antigenic	Predicts potentially antigenic regions of a protein sequence, based on occurrence frequencies of amino acid residue types in known epitopes.	[63]

5. TargetP1.1	Predicts the subcellular location of eukaryotic proteins based on the predicted presence of any of the N-terminal presequences: chloroplast transit peptide (**cTP**), mitochondrial targeting peptide (**mTP**) or secretory pathway signal peptide (**SP**).	[64]

5. SignalP 3.0	Predicts the presence and location of signal peptide cleavage sites in amino acid sequences from different organisms. The method incorporates a prediction of cleavage sites and a signal peptide/non-signal peptide prediction based on a combination of several artificial neural networks and hidden Markov models.	[65]

6. TMHMM Server v. 2.0	Predicts the transmembrane helices in proteins based on Hidden Markov Model.	[66]

7. Conserved Domain Database and Search Service, v2.22	The Database is a collection of multiple sequence alignments for ancient domains and full-length proteins. It is used to identify the conserved domains present in a protein query sequence.	[67]

8. BlastP	It uses the BLAST algorithm to compare an amino acid query sequence against a protein sequence database.	[68]

9. ABCPred	Predict *B cell epitope(s) *in an antigen sequence, using artificial neural network.	[69]

10. BcePred	Predicts linear B-cell epitopes, using physico-chemical properties.	[70]

11. Discotope 1.2	Predicts discontinuous B cell epitopes from protein three dimensional structures utilizing calculation of surface accessibility (estimated in terms of contact numbers) and a novel epitope propensity amino acid score.	[71]

12. BEPro	BEPro, uses a combination of amino-acid propensity scores and half sphere exposure values at multiple distances to achieve state-of-the-art performance.	[72]

13. Propred	Predicts MHC Class-II binding regions in an antigen sequence, using quantitative matrices derived from published literature. It assists in locating promiscous binding regions that are useful in selecting vaccine candidates.	[73]

14. IEDB-AR (Average Relative Binding Method)	Predicts IC(50) values allowing combination of searches involving different peptide sizes and alleles into a single global prediction.	[74,75]

15. Bimas	Ranks potential 8-mer, 9-mer, or 10-mer peptides based on a predicted half-time of dissociation to HLA class I molecules. The analysis is based on coefficient tables deduced from the published literature by Dr. Kenneth Parker, Children's Hospital Boston.	[76]

16. NetMHC 3.0	Predicts binding of peptides to a number of different HLA alleles using artificial neural networks (ANNs) and weight matrices.	[77]

17. AlgPred	Predicts allergens in query protein based on similarity to known epitopes, searching MEME/MAST allergen motifs using MAST and assign a protein allergen if it have any motif, search based on SVM modules and search with BLAST search against 2890 allergen-representative peptides obtained from Bjorklund et al 2005 and assign a protein allergen if it has a BLAST hit.	[78]

18. Allermatch	Predicts the potential allergenicity of proteins by bioinformatics approaches as recommended by the Codex alimentarius and FAO/WHO Expert consultation on allergenicity of foods derived through modern biotechnology.	[79]

#### Web Interface

The Webserver is built on Apache version 2.0. Server side scripting was done in PHP version 5.1.4. The programs running at back-end for compositional property calculation are written in C programming language. These C programs were compiled using the GNU gcc compiler 3.4.3 in the Itanium 2, 64-bit dual processor server running on Red Hat Linux Enterprise version 4. The client side scripting was prepared in HTML and AJAX. FungalRV can be best viewed with Mozilla Firefox and Internet Explorer. The database was developed using MySQL version 4.1.20 at back end and runs in Red Hat Enterprise Linux ES release 4. The database web interfaces have been developed in HTML and PHP 5.1.4, which dynamically execute the MySQL queries to fetch the stored data and is run through Apache2 server.

**Figure 3:**
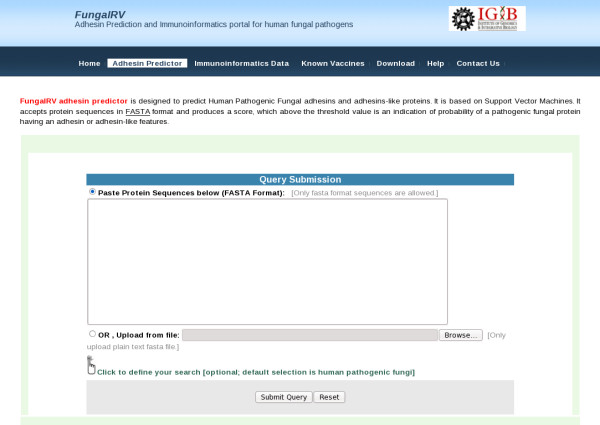
**FungalRV adhesin predictor Web site**. Users can paste or upload sequences in FASTA format for human pathogenic fungal adhesin and adhesin-like proteins prediction.

FungalRV web server has these tabs- "Adhesin Predictor", "Immunoinformatics Data", "Known Vaccines", "Download" and "Help". The "Adhesin Predictor" tab provides an interface where the users can paste or upload their query sequences and predict whether the protein sequence is a fungal adhesin (Figure 3). Users have the facility to set their own desired threshold cutoff value. The result can be exported as tab delimited text file by the users. The facility to search for fungal specific GPI pattern in the predicted adhesins and adhesin like proteins using fuzzpro program of EMBOSS has been provided [46,47]. Users also have been provided the facility to conduct BLAST search with human reference proteins.

**Figure 4:**
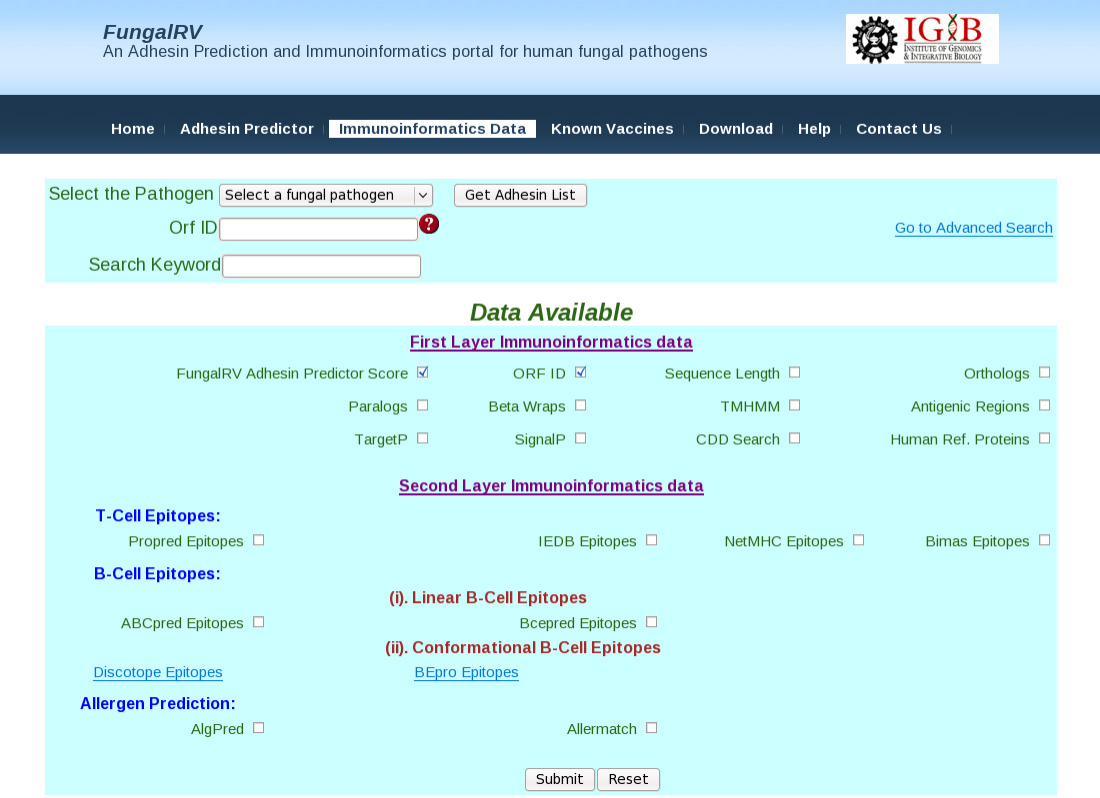
**FungalRV Immunoinformatics Web site**. Users can query FungalRV Immunoinformatics database for data useful from reverse vaccinology point of view corresponding to the predicted 307 adhesin and adhesin like proteins and known vaccine candidates.

On clicking the "Immunoinformatics Data" tab, users are directed to the FungalRV database of predicted fungal adhesins and adhesin like proteins (Figure 4). Here users can search the database for adhesin proteins and their attributes corresponding to one or more ORF identification tags of a species or against a specific keyword. Advanced search facility of predicted fungal adhesins is also provided where the results can be filtered on the basis of protein length, number of transmembrane spanning regions, localization and reliability class, presence or absence of betawraps, paralogs, hits to Conserved Domain Database and Human Reference proteins (retrieved from NCBI through ftp on 7 August, 2010). The results obtained can be exported by the user as a text file in both processes.

The "Known Vaccines" tab takes user to the page containing the list of known vaccine candidates provided in tabular form.

## Utility and Discussion

### Adhesin prediction for human fungal pathogens

#### User interface -

A user friendly interface was developed for using the "Fungal RV adhesin predictor" algorithm. Users can paste the sequence in FASTA format or even upload a file. A threshold of 0.511 was set as the optimal threshold (Figure 2). However, users can set a threshold of their own choice. The results are displayed in a colour coded tabular format. 'See additional file 2: Adhesins and adhesin like proteins predicted by "FungalRV adhesin predictor" in 8 human fungal pathogens'. Results can be exported in tab delimited text format.

Our algorithm "FungalRV adhesin predictor" predicted many cell surface GPI anchored proteins as novel adhesins from the 8 fungal pathogens. 'See additional file 3: GPI anchored proteins predicted as adhesin by FungalRV adhesin Predictor'. GPI anchor proteins in fungi are known to be either covalently incorporated into the cell wall network or remain attached to the plasma membrane. The predicted amino acid sequences of GPI proteins conform to a general pattern. Their N-termini has a hydrophobic signal sequence that directs the protein to the ER and their C-termini has a second hydrophobic domain, which is cleaved off and replaced with a GPI anchor (a preformed lipid in the membrane of the endoplasmic reticulum) by a transamidase enzyme complex. The GPI anchored proteins are linked to plasma membrane via this preformed GPI anchor [48]. These proteins may have roles in cell wall biosynthesis, cell wall remodeling, determining surface hydrophobicity and antigenicity and in adhesion and virulence [49,50].

In *C. albicans *"FungalRV adhesin predictor" predicted proteins proposed to be involved in the process of adhesion to host such as SUN41, IFF4 [51,52]. These proteins were not included in the training set due to absence of evidence on their direct involvement in adhesion process. However, their eventual prediction as adhesins by "FungalRV adhesin predictor" suggests their potential role in mediating adhesion. "FungalRV adhesin predictor" at optimal threshold of 0.511 predicts all the members of ALS and Hyr/iff (GPI family 17 and 18), proposed to be involved in modulating adhesion and biofilm formation in *C. albicans *[26]. The ALS family in *C. albicans *is characterized as the main class of adhesins [53,54]. Another protein RBT1 showing similarity to HWP1 and may have adhesion property [55] is also predicted by "FungalRV adhesin predictor".

In *C. glabrata*, several proteins showing similarity to flocculins and STA1 glucoamylase homologue of *S. cerevisiae *were predicted. 'See additional file 4: Predicted adhesins from *C. glabrata *with similarity to either flocculins or STA1'. The flocculins are involved in adhesion process in *S. cerevisiae *[56,57] and therefore it is probable that these proteins have functional similarity in their role as adhesins in *C. glabrata *as well. When compared to the predicted in-silico adhesins by Weig et al [25], the new release of *C. glabrata *proteome by Genolevures (Sep. 2009) retains 28 orfids of the 51 orfids predicted as adhesins in the older proteome release by Genolevures (June 2004). "FungalRV adhesin predictor" could predict 24 of the 28 in-silico predicted adhesins at optimal threshold value of 0.511. 'See additional file 5: "FungalRV adhesin predictor" scores of In-silico predicted adhesins by Weig et al'.

**Figure 5:**
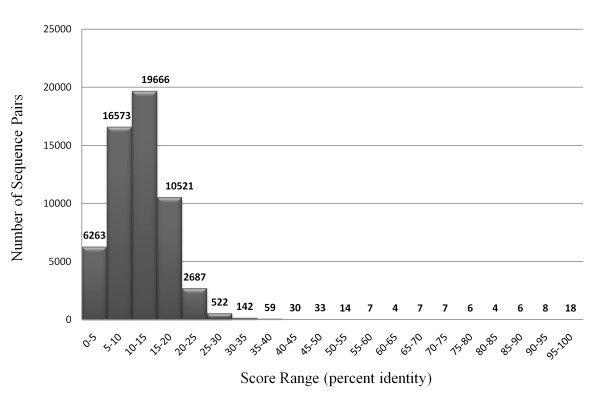
**Number of Sequence Pairs in the shown ClustalW score (percent Identity) ranges**. This graph was plotted for the 307 predicted fungal adhesins and adhesin like protein sequences from the selected eight human pathogenic fungal species. This data includes sequences from the training set.

ClustalW [58] analysis among the 307 predicted adhesin and adhesin like proteins obtained from "FungalRV adhesin predictor" run on entire proteomes of eight human pathogenic fungal species showed that most (99.65%) of the predicted adhesin sequence pairs have ClustalW score in the range of 0-35% (Figure 5). These data show that "FungalRV adhesin predictor" could predict adhesin sequences from diverse fungal pathogens thereby attesting its non-homology characteristic.

"FungalRV adhesin predictor" run on proteomes of some of the human pathogenic fungi with low incidence of occurrence- *Candida dubliniensis*, *Candida tropicalis, Candida parapsilosis, Candida lusitaniae *and *Candida guilliermondii *has been provided as supplementary data. 'See additional file 6: Adhesins and adhesin like proteins predicted by "FungalRV adhesin predictor" in other pathogenic fungi with low occurrence of incidence'.

Our algorithm FungalRV adhesin predictor uses highly accurate SVM models (greater than 99%) and therefore it achieves a good MCC of 0.8702 at a positive threshold of 0.511 in comparison to FAAPred [59], which uses SVM models of lower accuracy (86%) and achieves a MCC of 0.610 at a relatively high negative threshold of -0.8. FAAPred misses identifying integrins (a class of known adhesins) from *C. albicans *and *P. carinii *and in some cases identifies known adhesins with low score in the range (-0.06 to - 0.74) indicating low confidence predictions in contrast to our algorithm.

### Immunoinformatics Database

**Figure 6:**
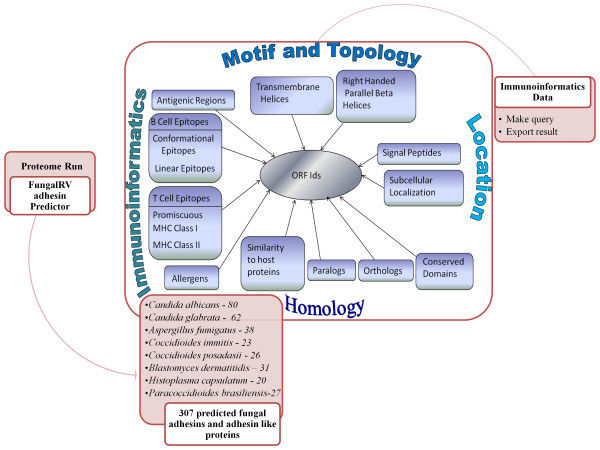
**Overall FungalRV Layout: The proteomes of eight human pathogenic fungal species listed in the diagram were run through "FungalRV adhesin predictor" obtaining a list of 307 fungal adhesins and adhesin like proteins**. The diagram provides a layout of analysis of the predicted proteins. All data are organized in relation to the primary key ORF ID. The analysis data obtained was arranged into FungalRV Database providing users' facility to query and export results into tab delimited text format.

The FungalRV immunoinformatics database houses immunoinformatics data on 307 predicted adhesins and adhesin like proteins obtained by "FungalRV adhesin predictor" run on entire proteomes of eight human pathogenic fungal species. This includes 80 from *C. albicans*, 62 from *C. glabrata*, 38 from *A. fumigatus*, 31 from *B. dermatitidis*, 27 from *P. brasiliensis*, 20 from *H. capsulatum*, 23 from *C. immitis *and 26 from *C. posadasii*. The database houses detailed information on proteins analysed through 18 algorithms important from the view of reverse vaccinology (Table 5) [60-79]. The analysis through these algorithms provide a broad range of information regarding Orthologs, Paralogs, BetaWraps, Localization, Transmembrane spanning regions, Signal Peptides, Conserved domains, similarity to Human Reference Proteins, T-cell epitopes, B-cell epitopes, Discotopes, and Allergen predictions. The overall layout of FungalRV is provided in Figure 6

First level of searching and retrieval of data is possible either through ORF ID or keywords. Multiple ORF IDs can be submitted using comma separation. Keywords can be used singly. If multiple keywords are used then the search is implemented using the AND Boolean. In the case of searching for epitope data, due to their huge size, data are conveniently retrieved in a singular mode for each ORF ID specifically. All data can be exported conveniently as a text file.

## Conclusion

A Web server aiding in novel human pathogenic fungal adhesin vaccine prediction and development has been prepared [80].

## Availability and Requirement

Sever can be accessed at http://fungalrv.igib.res.in. The server is best viewed with Explorer 8.0 or later and Mozilla firefox version 3.0 or later

## Competing interests

The authors declare that they have no competing interests.

## Authors' contributions

SR conceived the idea and provided guidance, suggestions, critical comments, and testing of FungalRV. RC prepared the positive and negative datasets and performed training to generate model classifiers. RC carried out testing, selection of the best models. RC collected immunoinformatics data, organized systematically, prepared the codes for FungalRV. FAA provided help with preparing the codes. MVR helped in system set up, maintenance and administration. SR and RC wrote the manuscript. All authors have read and approved the final manuscript.

## Authors' information

SR is a Bioinformatics scientist with focus on infectious diseases at the Institute of Genomics and Integrative Biology (CSIR), Delhi 110 007, India. RC is a Ph.D. student carrying out her thesis work at the Institute of Genomics and Integrative Biology (CSIR), Delhi 110 007, India. MVR is a systems scientist at the Institute of Genomics and Integrative Biology (CSIR), Delhi 110 007, India

## Supplementary Material

Additional file 1 **Pfam Domain Search Result of negative dataset**. The file presents Pfam domain search result on negative training set. Click here for file

Additional file 2 **Adhesins and adhesin like proteins predicted by FungalRV adhesin Predictor in 8 human fungal pathogens**. The file lists 307 adhesins and adhesin like proteins obtained by "FungalRV adhesin predictor" run on entire proteomes of eight human pathogenic fungal species along with their FungalRV adhesin predictor scores. Known adhesins are coloured in Green. Click here for file

Additional file 3 **GPI anchored proteins predicted as adhesin by FungalRV adhesin predictor**. FungalRV adhesin predictor predicted many cell surface GPI anchored proteins as novel adhesins. These proteins along with their FungalRV adhesin predictor score are listed in this file. Click here for file

Additional file 4 **Predicted adhesins from *Candida glabrata *with similarity to either flocculins or STA1**. Predicted adhesins from *Candida glabrata *with similarity to either flocculins or STA1 by "FungalRV adhesin predictor" along with their "FungalRV adhesin predictor" scores are listed in this file. Click here for file

Additional file 5 **"FungalRV adhesin predictor" scores of In-silico predicted adhesins by Weig et al**. "FungalRV adhesin predictor" scores of In-silico predicted adhesins by Weig et al. are listed in this file. Click here for file

Additional file 6 **Adhesins and adhesin like proteins predicted by "FungalRV adhesin predictor" in other pathogenic fungi with low incidence of occurrence**. The file lists adhesins and adhesin like proteins obtained by "FungalRV adhesin predictor" run on entire proteomes of some of the human pathogenic fungi with low incidence of occurrence along with their FungalRV adhesin predictor scores. Click here for file
